# How to fit allergen immunotherapy in the elderly

**DOI:** 10.1186/s12948-017-0075-2

**Published:** 2017-10-06

**Authors:** Erminia Ridolo, Anti Rogkakou, Maria Teresa Ventura, Irene Martignago, Cristoforo Incorvaia, Gabriele Di Lorenzo, Giovanni Passalacqua

**Affiliations:** 1 Medicine and Surgery Department, University of Parma, Via Gramsci 14, 43126 Parma, Italy; 2 Allergy and Respiratory Diseases Clinic, DIMI, University of Genoa, IRCCS AOU San Martino-IST, Genoa, Italy; 3 Department of Interdisciplinary Medicine, University of Bari, Bari, Italy; 4 Cardiac/Pulmonary Rehabilitation, ASST Pini/CTO, Milan, Italy; 5 Dipartimento BioMedico di Medicina Interna e Specialistica (Di.Bi.M.I.S), Università di Palermo, Palermo, Italy

**Keywords:** Allergy, Elderly, Immunosenescence, Allergen immunotherapy

## Abstract

Asthma, allergic rhinitis (AR) and atopic dermatitis are very common in young people, but in the latest decades it was increasingly recognized that also individuals of higher ages, including the population over 65 years, are concerned. Actually, it is now acknowledged the aging does not considerably alter the immune response to allergens. Allergen immunotherapy (AIT) is the only treatment that works on the causes of allergy, but elderly people are commonly excluded from AIT, except the cases of insect sting allergy. A number of recent studies showed that aged individuals also successfully respond to AIT for respiratory allergy. Therefore, there is no reason to exclude elder patients from AIT. Anyhow, clinical conditions that are considered absolute or relative contraindications are quite frequent in this aged population, thus the risk/benefit ratio must be carefully evaluated for each patient, taking into account that the more frequent occurrence of co-morbidities and the consequent need of daily-based multidrug regimen can favor adverse effects. An important issue concern the ability of AIT, and particularly of sublingual immunotherapy, to significantly improve the quality of life, that often is particularly impaired in the elderly, reducing symptoms and drugs consumption.

## Background

Asthma, allergic rhinitis (AR) and other atopic diseases (e.g. food allergy or atopic dermatitis) are very common in young people, but it is now recognized they can also affect individuals of higher ages. For instance, AR was reported to affect up to 5–10% of the population over 65 years [1]. However, a global decline of the prevalence of allergic disorders in elderly, at least on epidemiological basis has been repeatedly claimed. This might be ascribed to the expected decrease in serum IgE antibodies due to an unbalance of cytokines and soluble factors involved in its production. However, in studying non-allergic individuals aged 20–93 years, assessing serum IgE, sCD23 and Th2 type cytokine production, IgE levels were not significantly different between young and old subjects [2]. This was confirmed in another similar study [3]. These results suggest that the type 2 cytokine pattern is not necessarily defective in older age. Data also confirmed that IL-13, a key cytokine in IgE regulation, is not impaired in old subjects. Although IL-4 has been considered the most critical cytokine linked to allergic responses and immunity against parasites, recent observations indicate that IL-13 has equal or even greater importance in those processes. IL-4 and IL-13 share several functional properties, however IL-13 can independently induce class switching and IgE secretion from human B cells. In addition, IL-13 enhances expression of CD23 and of the major histocompatibility complex class II antigens, and may act as a monocyte chemoattractant [4, 5]. The clinical findings indicating that the allergic reactivity could decrease with age [6, 7] make ageing an interesting in vivo model to study the waning of allergic response. Yet, there is not sufficient information to establish that the frequency of onset of allergic symptoms, as well as their severity, declines with aging. According to the aforementioned aspects, the clinical use of allergen specific immunotherapy (AIT) has been so far considered of little interest in the elderly, since AIT is commonly used in young people, with a well-defined TH2-high immunological phenotype. We review herein the main immunologic aspects of aging, according to the presence of allergic diseases, and the current evidence on the use of AIT in elderly patients.

## Aging and the immune system

Immunosenescence is a complex process in which genetic and environmental factors are involved, characterized by a “remodeling” more than a “decline” of the immune system, and associated to various factors: oxidative stress, apoptotic phenomena, increased production of pro-inflammatory lymphokines [8]. This results in a state of chronic, low-grade, inflammation described by some authors as “inflamm-aging” [9]. The cells of the immune system are constantly renewed by the hematopoietic stem cells, but during senescence this activity declines and the overall amount of hematopoietic tissue decreases. This event also seems related to the shortening of telomeres [10]. Thymic involution process in the elderly goes along with the reduction of naϊve T cells, although the total number of T cells does not undergo large displacements due to the increase in population of non-regulatory cells CD8+ CD45RO+ CD25+, that represent a promising biomarker of immune competence in old age. In the elderly a reduced response of T cells to growth factors and specific antigens can be documented [11]. The compartment of B cells is also affected by ageing and this influences the efficiency of cooperation between T and B lymphocytes. Even if the total B cells does not change, the number of memory B lymphocytes, becoming less sensitive to apoptosis, increases. At the same time, the naϊve B cells are reduced [12]. All these changes may result in an enhancement of monoclonal serum immunoglobulins. The role of regulatory T cells (Treg), a cell subsets with regulatory function, including CD4+ and CD8+ Treg, deserves special attention in the elderly. CD4+ Tregs are involved in suppressing the activation, proliferation, and cytokine production, as well as the function of dendritic cells. The Tregs are characterized by the expression of IL-2R α-chain (CD25) and CTLA-4. The transcription factor FoxP3 (Forkhead box P3) plays an important role in the function of Tregs. The naturally occurring CD4+ and CD8+ Treg increase in the elderly and have equivalent expression of CTLA-4 and equivalent regulatory function with cytokine production after polyclonal T cell stimulation compared to younger subjects, while the number of inducible CD4+ and CD8+ Treg decreases with advancing years [13]. The relevance of these cells in the elderly is not yet fully clarified in relation to immunosenescence. In particular, the Tregs induce down-regulation of mast cells, basophils and eosinophils through the production of IL-10 and TGF-β and may also induce reduction of Th2 cells and their cytokines. In turn, IL-10 and TGF-β elicit the suppression of IgE by B lymphocytes and, in particular, IL-10 causes the switch towards IgG4 producing plasma cells and consequently to an increased synthesis of allergen-specific IgG4 isotype blocking antibodies [14, 15]. Recently, it was identified in the senescence an immunological risk phenotype (IRP) characterized by inversion of the CD4/CD8 ratio, increase in CD8 + CD28 ^−^ lymphocyte population, identified as memory/effector cells and reduction of B lymphocytes. In addition, patients with IRP manifested a marked serum positivity for cytomegalovirus and the increase of proinflammatory cytokines, including in particular IL-6, IL-1β, tumor necrosis factor (TNF)-α and acute phase proteins [16]. An interesting research in elderly patients has shown not only an enhancement of the type 1 cytokines such as IL-1, interferon (IFN)-γ and TNF-α, whose role is of great importance for the increase of cancer and chronic infections, but also of type 2 such as IL-4, IL-5, IL-10, which constitutes the substrate for the development of allergic diseases [2]. In particular, in cytokine cultures stimulated with PHA, the production of T-helper 2 cytokines does not appear to be significant different in young and elderly subjects. As it is well known that in allergic patients there is an overproduction of Th2 cytokines, that can induce the isotopic switch of B cells toward IgE isotype immunoglobulins and recall on-site cells responsible for the allergic inflammation (mast cells and eosinophils), the authors concluded that there is no valid justification for the statement that allergic diseases decline in the elderly. Moreover, the same authors showed that IL-13, that plays a key role in the regulation of IgE, is not affected in the elderly [2] thus confirming that the occurrence of allergic reactions in the elderly is supported by high serum levels of IgE and type 2 cytokines. However, data on the production of IgE in the literature are contradictory. In fact, in opposition to the claim that the levels are reduced in the elderly [17], in the most recent literature there is increasing evidence that in the allergic disease IgE levels are perfectly comparable to those found in young subjects [2, 18]. We must remember that an efficient IgE response requires both the presence of IL-4 or IL-13 and also a physical interaction between T and B-cells, involving a number of surface and adhesion molecules [19, 20], that include the CD40/CD40L interaction that is defective in old subjects [21]. However, these studies analyzed young and old non-allergic individuals, while it would be of greater interest to compare these parameters in allergic young and old subjects.

Alterations of innate immunity have an essential role and the studies on the issue have identified a separate trend in the field of immunogerontology, beginning with the reduction of epithelial barriers in the skin and mucous membranes, both at respiratory and gastrointestinal tract [22]. As regards the immunoglobulins of IgA type at the mucosal level, significant changes can be found with a marked increase of monomeric IgA1, both in serum and in saliva. Some changes, especially in the functional aspects related to chemotaxis, phagocytosis and oxidative burst are shown both in the compartment of neutrophils and macrophages. In addition, it is possible to observe reductions in the level of macrophage-derived chemokines, including macrophage inflammatory protein (MIP)-1α, MIP-1β and eotaxin. The natural killer (NK) cells do not decrease in number, indeed their absolute number was found to increase in geriatric patients. However, the reduced production of IL-2, of critical importance in the processes of lymphocyte activation and killing, induces a deficiency of cytotoxic function in which the numerical increase may occur for a mere compensatory mechanism [23]. Also, dendritic cells play an important role, both in the innate and adaptive immunity. These cells have the role to start the immune response with the function of antigen presenting cells (APC), becoming able to process the antigen and to secrete cytokines. The prolonged administration of allergen as occurs during AIT promotes the production of high levels of IL-12 and IL-27, favouring allergen-specific switches from the Th2 to the Th1 and Th17 phenotype and the expression of NK and NKT cells which in turn induce the increased levels of IFN-γ and activation of APC [24]. The role of vitamins and trace elements, including iron and zinc should be also considered, especially in the light of the frequent conditions of malnutrition in geriatric patients. Vitamin D, and especially its active form, is very important in both innate and adaptive immunity. In particular calcitriol is able to act on APCs and on T lymphocytes by determining inhibition of inflammatory responses through induction of T reg [25].

## Allergen sensitizations and AIT in the elderly

Elderly spend most of their time indoors and consequently the most frequent allergen sensitizations are towards indoor allergens, such as house dust mites, cockroaches, furred pets [26]. AIT is an important guideline-approved therapy for AR and asthma, which can modify the disease’s natural history, with a long lasting effect on symptoms after its cessation [27]. Indeed, IgE positivities reveal sensitization, but clinical allergy has to be confirmed on the ground of evidence of a relationship between results of IgE test and occurrence of symptoms from exposure to the specific allergen, as well as by demonstration of concomitant inflammation in the airways as assessed by the presence of eosinophils in nasal smears and increase of FeNO [28]. These investigations can be easily performed and are mandatory for the AIT indication in these conditions. A large number of studies confirmed the efficacy and safety of AIT in AR and asthma [29], which is especially related to the mechanism of action itself [15]. Of note, only few studies were designed for AIT in the elderly. Therefore, specific indications and contraindications for AIT in old people are not yet available. Currently, allergists can rely, their everyday practice only on the general guidelines also for the elderly.

The GA^2^LEN/EAACI pocket guide on AIT for AR and asthma points out that there is not an upper age limit for prescribing AIT [28], as also suggested by the recent EAACI (European Academy of Allergy and Clinical Immunology) position paper on the clinical contraindications to AIT [30], that gave no indication on the upper age limit. In the mentioned document, the only absolute contraindications to AIT were the presence of uncontrolled asthma, autoimmune disorders not responding to therapy, active immune-deficiency and active malignancies. These chronic diseases are frequent among elderly and it is, therefore, warranted to collect a detailed medical history before prescribing AIT. Uncontrolled asthma can depend on an inappropriate therapy and/or poor compliance by the patient. If adequate adjustment of therapy and careful education of the patient allows a good control of asthma symptoms, AIT can be administered.

Autoimmune diseases in remission phase are considered a relative contraindication. Concerning hymenoptera venom immunotherapy (VIT), the position paper states “VIT is a highly advised option in high-risk venom allergic patients” [30]. There are few data on the risk of developing autoimmune diseases. Only a Danish observational study on 18,841 patients treated with subcutaneous immunotherapy (SCIT) vs. 428,484 controls treated with drug therapy for over 10 years found that there was a lower incidence of autoimmune diseases in patients undergoing subcutaneous immunotherapy (SCIT) [31]. In a recent survey among American allergists, it was observed that AIT (VIT and pollen SCIT) performed in patients with stable autoimmune diseases had an high safety profile [32, 33]. Concerning cancer, there is currently no evidence of a new onset or relapse when administering AIT. The possibility that the immunomodulation stimulated with AIT may have a negative effect on the natural history of cancer is the real concern about prescribing AIT [34]. A study from Austria study analyzed the use of AIT in six patients with cancer [35]. One of them received SCIT after 14 years of melanoma diagnosis without presenting relapse of the malignancy during AIT for house dust mites, while another patient interrupted SCIT for grass-pollen because the diagnosis was made few months after AIT initiation. Another patient undergoing VIT had a relapse of breast cancer 3 months after having reached the maintenance dose of immunotherapy, but she ended the 5 years course of VIT while maintaining the neoplastic disease under control. No immunologic mechanisms able to account for a negative effect of AIT on cancer were detected thus far.

Cardiovascular diseases, such as unstable angina, recent myocardial infarction, significant arrhythmia, and uncontrolled systemic hypertension, are very common in old people. The EAACI document considers them as a relative contraindication for inhalant AIT and advises to accurately evaluate the current status of the disease before starting AIT [30]. In fact, the Danish observational study cited above pointed out that SCIT was associated with a lower incidence of acute myocardial infarction and a lower risk of developing ischemic heart disease, compared to medical conventional therapy [31]. Also the presence of chronic pulmonary diseases (not only asthma) that reduce lung function must be evaluated in elderly patients before administering AIT [36].

A daily-based multidrug regimen is widely diffuse in population over 60 years and antihypertensive drugs are among the medications most frequently taken. In everyday practice the allergist often encounters patients who take β-blockers and ACE-inhibitors. In a study on murine models, Nassiri et al. stated that the use of β-blockers slightly aggravates an anaphylactic reaction, while the combined therapy with β-blockers and ACE-inhibitors synergistically could worsen anaphylaxis, due to their action on reducing the mast-cell activation threshold [37]. Clinically, β-blockers use is considered a relative contraindication for VIT, because the block of β1 and β2-receptors decreases the epinephrine action in case of anaphylaxis. However, β-blockers may be replaced by other kind of drugs in case of hypertension but not in case of arrhythmia or ischemic disease. Also ACE-inhibitors use is controversial in patients undergoing VIT, but do not represent a contraindication for inhalant AIT [30]. Importantly, the possible contraindications to use β-blockers and ACE-inhibitors concern only SCIT and not sublingual immunotherapy (SLIT).

Other chronic diseases are common in the elderly and must be evaluated before prescribing AIT. Patients with psychiatric or mental disorders could be not enough compliant or can be unable to report early symptoms of AIT side-effects. Concerning drug treatment of such disorders, the use of monoamine oxidase inhibitors is not a contraindication for AIT, but it must be considered that their action combined with epinephrine could cause severe hypertension and tachycardia [38].

On the other hand, AIT has an important outcome in reducing the risk of side effects to anti-allergic drugs, such as oral corticosteroids (diabetes, osteoporosis, hypertension, etc.), and anti-histamines (sedation, anti-cholinergic effects).

According to the available data there are not real limitations in administering AIT to elderly people if we consider only age as a limit, but thus far studies on AIT in the elderly are rare, because they are often excluded (mainly for concomitant diseases requiring daily drug-regimen) or present in a small number. Another limit concern defining the elderly age: in developed countries people over 65 of age is usually considered as elderly [39], but often the inclusion age criteria to participate to an AIT trial is 60 years or less. The first study about efficacy and safety of AIT in old patients was performed in 1993 by Armentia et al. [40] on 22 geriatric patients treated with AIT, in whom a significant clinical improvement and a decrease in drug use was achieved. In 2000 Eidelman et al. demonstrated for the first time that AIT was effective in elderly as much as in the younger, treating with SCIT otherwise healthy patients over 60 years of age. In this case the authors chose, as control group, patients with similar allergic history before AIT, but aged ≤ 60 years, obtaining a similar good response in both groups of patients [41]. In 2004 Asero investigated whether SCIT could be safely performed in elderly sensitized to seasonal allergens (birch and ragweed); 39 subjects (aged between 55 and 70 years) with a disease’s duration of less than 10 years were included. Patients underwent AIT for 1–5 years, achieving a lower use of cetirizine (p < 0.001) and/or salbutamol (p < 0.05), compared to controls [42].

In recent years an increasing number of studies addressed the safety and efficacy of SLIT [43]. The first study investigating SLIT in elderly population was conducted in 2008 by Marogna et al. In the overall study population of 167 patients (including subjects aged 18–65 years) with AR and mild asthma due to dust mite allergy, 40 patients were aged more than 55 years [44]. The patients underwent SLIT with a dust mite extract and the results were compared to a control group treated with standard drug treatment. The end points were symptoms and drug scores every month, respiratory function tests, metacholine challenge and eosinophils counts at the start and at the end of the study. The authors found in the SLIT group an improvement in all examined variables (p < 0.001), achieving also a lower number of new allergen sensitizations (p = 0.03), compared to the control group. The results were similar in both young and elderly patients as long as the disease had started fairly recently. However, it is needed to underline that no elderly subject was included, according to the common accepted limit age stated above [39]. The same limit is present in the trial by Baptistella et al. who treated 104 patients with AR over 55 of age (55–74 years) with SLIT for dust mites for 1 year, but there was no information on the number of patients aged more than 65 years [45]. Moreover, patients undergoing any kind of treatment for any comorbidity were excluded. The authors demonstrated the efficacy and the safety of SLT in their patients, with clinical improvement on rhinitis symptoms and absence of side effects. The critical issue regarding these SLIT studies is that they were not blinded.

The first two double-blind placebo-controlled trials were performed recently on a population over 60 years of age. In the initial trial, Bozek et al. recruited 106 patients between 60 and 75 years of age with demonstrated AR due to dust mites [46]; 51 subjects underwent SLIT and the other 57 were included in the placebo group. After 3 years of treatment the patients treated with SLIT had a significant improvement of 44% (p < 0.05) in symptoms and of 35% for the medication scores (p < 0.05). No side effect was reported. The same authors examined the safety and the efficacy of SLIT for grass pollen allergy in a population aged over 60 years [47]; 78 subjects between 60 and 70 years of age were included in the trial and divided in two groups: 41 patients were treated with SLIT and 37 were in the placebo group. At the end of AIT (3 years), the total nasal symptom score and the total medication score in the SLIT group improved significantly, obtaining a decrease of 64 and 51%, respectively (p < 0.05 for both). Only three patients had common local side effects, such as oral itching and facial flushing, while no systemic reaction was observed. Recently, these authors also investigated the safety and efficacy of pre-seasonal SCIT for grass pollen allergy in 62 patients older than 65 years with seasonal AR. It was demonstrated that the total nasal symptom score decreased by 76% and that the total medication score of the active group decreased by a maximum of 62% in the active group after 3 years of AIT. Moreover, no adverse systemic reactions were reported during therapy [48]. Studies about AIT in elderly are summarized in Table 1.

**Table 1:** AIT studies in elderly

Study	N. patients	AR/AA	Allergen	SCIT/SLIT	Length (years)
Armentia et al. [40]	22	NA	NA	SCIT	1
Eidelman et al. [41]	26	AR and AA	NA	SCIT	NA
Asero [42]	39	AR and AA	Birch and ragweed	SCIT	1–5
Marogna et al. [44]	40	AR and AA	HDM	SLIT	NA
Baptistella et al. [45]	104	AR and AA	HDM	SLIT	1
Bozek et al. [46]	106	AR	HDM	SLIT	3
Bozek et al. [47]	78	AR	Grass pollen	SLIT	3
Bozek et al. [48]	62	AR	Grass pollen	SLIT	3

*AR* allergic rhinitis, *AA* allergic asthma, *SCIT* subcutaneous immunotherapy, *SLIT* sublingual immunotherapy, *HDM* house dust mites, *N.A.* not available data

## Conclusions

The older age per se does not preclude the prescription of AIT, that should be considered in elderly individuals when there is a clear indication. Anyhow, clinical conditions that are considered absolute or relative contraindications are quite frequent in this age population and can interfere with AIT effectiveness and safety. The risk/benefit ratio must be carefully evaluated for each patient, taking into account that in the elderly the frequent occurrence of co-morbidities and the consequent need of daily-based multidrug regimen can favor adverse effects as well as drug interactions [49]. An important issue concern the ability of AIT, particularly SLIT, to significantly improve the quality of life, reducing symptoms and drugs consumption [43]. Actually, a study stated that in patients with AR the impairment of quality of life is significantly higher in elderly than in young people [50]. In older patients, this may result in altered cognitive functions giving rise to a vicious circle with unremitting worsening, that warrants to be broken by treatment with significant effectiveness and particularly with persistence over time also following its stopping, thus ensuring to patients a good control of allergic symptoms with no or minimal use of symptomatic drugs. Such requirement is fulfilled by the therapeutic performances of AIT.

## Abbreviations

AIT: allergen specific immunotherapy
APC: antigen presenting cells
AR: allergic rhinitis
EAACI: European Academy of Allergy and Clinical Immunology
FeNO: fractional exhaled nitric oxide
IFN: interferon
IRP: immunological risk phenotype
MIP: macrophage inflammatory protein
NK: natural killer
SLIT: sublingual immunotherapy
SCIT: subcutaneous immunotherapy
TNF: tumor necrosis factor
Treg: regulatory T cells
VIT: hymenoptera venom immunotherapy

## References

[1] Cardona V, Guilarte M, Luengo O (2011). Allergic diseases in the elderly. Clin Transl Allergy.

[2] Di Lorenzo G, Pacor ML, Esposito Pellitteri M, Listì F, Colombo A, Candore G, Mansueto P, Lo Bianco C, Ditta V, Battista Rini G, Caruso C (2003). A study of age-related IgE pathophysiological changes. Mech Ageing Dev.

[3] Moro-García MA, Alonso-Arias R, López-Larrea C (2012). Molecular mechanisms involved in the aging of the T-cell immune response. Curr Genomics.

[4] Caramori G, Groneberg D, Ito K, Casolari P, Adcock IM, Papi A (2008). New drugs targeting Th2 lymphocytes in asthma. J Occup Med Toxicol..

[5] Walford HH, Doherty TA (2013). STAT6 and lung inflammation. JAKSTAT..

[6] Di Lorenzo G, Leto-Barone MS, La Piana S, Ditta V, Di Fede G, Rini GB (2013). Clinical course of rhinitis and changes in vivo and in vitro of allergic parameters in elderly patients: a long-term follow-up study. Clin Exp Med..

[7] Marone G, Poto S, Giugliano R, Bonini S (1985). Studies on human basophil releasability. Int Arch Allergy Appl Immunol..

[8] Franceschi C, Capri M, Monti D, Giunta S, Olivieri F, Sevini F, Panourgia MP, Invidia L, Celani L, Scurti M, Cevenini E, Castellani GC, Salvioli S (2007). Inflammaging and anti-inflammaging: a systemic perspective on aging and longevity emerged from studies in humans. Mech Ageing Dev.

[9] Cavanagh MM, Weyand CM, Goronzy JJ (2012). Chronic inflammation and aging: DNA damage tips the balance. Curr Opin Immunol.

[10] Maicher A, Kastner L, Dees M, Luke B (2012). Deregulated telomere transcription causes replication-dependent telomere shortening and promotes cellular senescence. Nucleic Acids Res..

[11] Schwaiger S, Wolf AM, Robatsher P, Jenewein B, Grubeck-Loebenstein B (2003). Il-4-producing CD8+ T cells with a CD62L++(bright) phenotype accumulate in a subgroup of older adults and are associated with the maintenance of intact humoral immunity in old age. J Immunol..

[12] Weksler ME, Szabo P (2000). The effect of age on the B-cell repertoire. J Clin Immunol.

[13] Jagger A, Shimojima Y, Goronzy JJ, Weyand C (2014). Regulatory T cells and immune aging process. A mini review. Gerontology.

[14] Radulovic S, Jacobson MR, Durham SR, Nouri-Aria KT (2009). Grass pollen immunotherapy induces Foxp3-expressing CD4+ CD25+ cells in the nasal mucosa. J Allergy Clin Immunol.

[15] Akdis CA, Akdis M (2014). Mechanisms of immune tolerance to allergens: role of IL-10 and T regs. J Clin Invest..

[16] Wikby A, Ferguson F, Forsey R, Thompson J, Strindhall J, Löfgren S, Nilsson BO, Ernerudh J, Pawelec G, Johansson B (2005). An immune risk phenotype, cognitive impairment, and survival in very late life: impact of allostatic load in Swedish octogenarian and nonagenarian humans. J Gerontol A Biol Sci Med Sci.

[17] Jakola DR, Pierson-Mullant LK, Daniels LR, Rosenberg AQ, Blumental R (2003). Robustness into advanced age of atopy-specific mechanism in atopy-prone family. A Boil Sci Med Sci.

[18] Ventura MT, Gelardi M, D’Amato A, Buquicchio R, Tummolo R, Misciagna G, Canonica GW, Passalacqua G (2012). Clinical and cytologic characteristics of allergic rhinitis in elderly patients. Ann Allergy Asthma Immunol.

[19] Hänel KH, Cornelissen C, Lüscher B, Baron JM (2013). Cytokines and the skin barrier. Int J Mol Sci.

[20] Bunyavanich S, Rifas-Shiman SL, Platts-Mills TA, Workman L, Sordillo JE, Camargo CA, Gillman MW, Gold DR, Litonjua AA (2014). Peanut, milk, and wheat intake during pregnancy is associated with reduced allergy and asthma in children. J Allergy Clin Immunol..

[21] Blaeser A, McGlauchlen K, Vogel LA (2008). Aged B lymphocytes retain their ability to express surface markers but are dysfunctional in their proliferative capability during early activation events. Immun Ageing..

[22] Gomez CR, Boehmer ED, Kovacs EJ (2005). The aging innate immune system. Curr Opin Immunol.

[23] Borrego F, Alonso MC, Galiani MD, Carracedo J, Ramirez R, Ostos B, Peña J, Solana R (1999). NK phenotypic markers and IL2 response in NK cells from elderly people. Exp Gerontol.

[24] Maggi E (2010). T cell response induced by allergen specific therapy. Clin Exp Immunol.

[25] Chambers ES, Hawrylowicz CM (2011). The impact of vitamin D on regulatory T cells. Curr Allergy Asthma Rep..

[26] Yáñez A, Cho SH, Soriano JB (2014). Asthma in the elderly: what we know and what we have yet to know. World Allergy Organ J.

[27] Demoly P, Calderon MA, Casale TB, Malling HJ, Wahn U (2015). The value of pre- and co-seasonal sublingual immunotherapy in pollen-induced allergic rhinoconjunctivitis. Clin Transl Allergy..

[28] Zuberbier T, Bachert C, Bousquet PJ, Passalacqua G, Walter Canonica G, Merk H, Worm M, Wahn U, Bousquet J (2010). GA2LEN/EAACI pocket guide for allergen-specific immunotherapy for allergic rhinitis and asthma. Allergy.

[29] Passalacqua G, Canonica GWC (2016). Allergen immunotherapy: history and future developments. Immunol Allergy Clin North Am..

[30] Pitsios C, Demoly P, Bilò MB (2015). Clinical contraindications to allergen immunotherapy: an EAACI position paper. Allergy.

[31] Linneberg A, Jacobsen RK, Jespersen L, Abildstrøm SZ (2012). Association of subcutaneous allergen-specific immunotherapy with incidence of autoimmune disease, ischemic heart disease, and mortality. J Allergy Clin Immunol..

[32] Calabria CW, Hauswirth DW, Rank M, Sher L, Larenas-Linnemann D (2017). American Academy of Asthma, Allergy & Immunology membership experience with venom immunotherapy in chronic medical conditions and pregnancy, and in young children. Allergy Asthma Proc..

[33] Larenas-Linnemann DE, Hauswirth DW, Calabria CW, Sher LD, Rank MA (2016). American Academy of Allergy, Asthma & Immunology membership experience with allergen immunotherapy safety in patients with specific medical conditions. Allergy Asthma Proc..

[34] Ridolo E, Montagni M, Bonzano L, Senna G, Incorvaia C (2014). Arguing the misconceptions in allergen-specific immunotherapy. Immunotherapy.

[35] Wöhrl S, Kinaciyan T, Jalili A, Stingl G, Moritz KB (2011). Malignancy and specific allergen immunotherapy: the results of a case series. Int Arch Allergy Immunol..

[36] Scichilone N, Ventura MT, Bonini M (2015). Choosing wisely: practical considerations on treatment efficacy and safety of asthma in the elderly. Clin Mol Allergy.

[37] Nassiri M, Babina M, Dölle S (2015). Ramipril and metoprolol intake aggravate human and murine anaphylaxis: evidence for direct mast cell priming. J. Allergy Clin Immunol..

[38] Livingston MG, Livingston HM (1996). Monoamine oxidase inhibitors. An update on drug interactions. Drug Saf.

[39] Bernabei R, Gray L, Hirdes J, Halter JB, Ouslander JG, Tinetti ME, Studenski S, High KP, Asthana S (2009). International gerontology. Hazzard’s geriatric medicine and gerontology.

[40] Armentia A, Fernández A, Tapias JA (1993). Immunotherapy with allergenic extracts in geriatric patients: evaluation of effectiveness and safety. Allergol Immunopathol (Madr).

[41] Eidelman F, Darxentas N (2000). Efficacy of allergy immunotherapy in the elderly. J Allergy Clin Immunol.

[42] Asero R (2004). Efficacy of injection immunotherapy with ragweed and birch pollen in elderly patients. Int Arch Allergy Immunol..

[43] Canonica GW, Cox L, Pawankar R (2014). Sublingual immunotherapy: World Allergy Organization position paper 2013 update. World Allergy Organ J.

[44] Marogna M, Bruno ME, Massolo A, Falagiani P (2008). Sublingual immunotherapy for allergic respiratory disease in elderly patients: a retrospective study. Eur Ann Allergy Clin Immunol..

[45] Baptistella E, Maniglia S, Malucelli DA (2013). Allergen-specific immunotherapy in patients 55 years and older: results and review of literature. Int Arch Otorhinolaryngol.

[46] Bozek A, Ignasiak B, Filipowska B, Jarzab J (2013). House dust mite sublingual immunotherapy: a double-blind, placebocontrolled study in elderly patients with allergic rhinitis. Clin Exp Allergy.

[47] Bozek A, Kolodziejczyk K, Warkocka-Szoltysek B, Jarzab J (2014). Grass pollen sublingual immunotherapy: a double-blind, placebo-controlled study in elderly patients with seasonal allergic rhinitis. J Am J Rhinol Allergy..

[48] Bozek A, Kolodziejczyk K, Krajewska-Wojtys A, Jarbaz J (2016). Pre-seasonal, subcutaneous immunotherapy: a double-blinded, placebo-controlled study in elderly patients with an allergy to grass. Ann Allergy Asthma Immunol.

[49] Ridolo E, Caminati M, Martignago I (2016). Allergic rhinitis: pharmacotherapy in pregnancy and old age. Expert Rev Clin Pharmacol..

[50] Ventura MT, Gelardi M, D’Amato A, Buquicchio R, Tummolo R, Misciagna G (2012). Clinical and cytologic characteristics of allergic rhinitis in elderly patients. Ann Allergy Asthma Immunol.

